# Biomarkers in ALS trials: from discovery to clinical utility

**DOI:** 10.3389/fnins.2025.1636303

**Published:** 2025-11-28

**Authors:** Triparna Roy, Ammar Al-Chalabi, Alfredo Iacoangeli, Ahmad Al Khleifat

**Affiliations:** 1 King’s College London, Institute of Psychiatry, Psychology & Neuroscience, London, United Kingdom; 2 King’s College Hospital, NHS Foundation Trust, London, United Kingdom; 3 Department of Biostatistics and Health Informatics, King’s College London, London, United Kingdom

**Keywords:** ALS (amyotrophic lateral sclerosis), MND (motor neuron disease), biomarker, clinical trial (CT), neurodegenerative disease, ALS, neurofilament light chain (NfL), precision medicine

## Abstract

**Introduction:**

Motor neuron disease (MND), also known as amyotrophic lateral sclerosis (ALS), is a progressive neurodegenerative disorder characterized by motor neuron degeneration, leading to muscle weakness, paralysis, and eventual respiratory failure. Despite advances in understanding its pathology, effective therapies remain limited, underscoring the need for reliable biomarkers to aid early diagnosis, monitor disease progression, and optimize clinical trials. This systematic review explores the role of biomarkers in ALS, focusing on their application in clinical trials to accelerate therapeutic development and enhance patient care.

**Methods:**

A comprehensive search of PubMed, EMBASE, MedLine, and Google Scholar identified 93 studies investigating various biomarkers, including neurofilament light chain (NFL), inflammatory markers, genetic markers like *SOD1* and *C9orf72*, and imaging modalities.

**Results:**

NFL emerged as a robust biomarker, strongly correlating with disease progression and therapeutic response, and was frequently used in trials like RESCUE-ALS and CENTAUR. Genetic biomarkers, such as *C9orf72* and *SOD1* mutations, provided insights into ALS mechanisms and informed targeted therapeutic approaches. Emerging biomarkers, such as retroviral elements, show potential but require further validation. Included studies span key trials such as Lighthouse-II, MIROCALS, and MND-SMART.

**Discussion:**

This systematic review evaluates which biomarkers are currently validated for monitoring disease progression and therapeutic response in ALS clinical trials, including protein, genetic, inflammatory, metabolic, and imaging markers. It also highlights the critical role of biomarkers in advancing MND clinical trials by enabling adaptive trial designs, patient stratification, and the use of surrogate endpoints, thereby reducing trial duration and improving efficiency. The review also highlights the translational gap between biomarker discovery and clinical application, emphasizing their potential to optimize trial design and patient stratification. While biomarkers like NFL have transformed trial methodologies, challenges such as disease specificity and inter-patient heterogeneity persist. Future efforts should focus on multimodal biomarker approaches to achieve comprehensive disease assessment and advance personalized therapeutic strategies, ultimately improving outcomes for patients with MND.

## Introduction

Motor neuron disease (MND), often referred to as amyotrophic lateral sclerosis (ALS), is a progressive neurodegenerative disorder characterized by the degeneration of upper and lower motor neurons in the spinal cord, brainstem, and motor cortex (Brotman et al., 2024; Hardiman et al., 2017). This leads to progressive muscle weakness, paralysis, and ultimately respiratory failure, typically within 3–5 years of diagnosis (Hardiman et al., 2017). Despite growing insights into its molecular pathology, ALS remains incurable; current treatments such as riluzole and edaravone offer only modest survival benefits (Hinchcliffe and Smith, 2017). The rapid progression and clinical heterogeneity of ALS underscore the urgent need for reliable biomarkers to aid early diagnosis, monitor disease progression, and improve clinical trial efficiency. Despite extensive biomarker discovery in ALS, translation into clinical practice and clinical trial application remains limited. Challenges include variability across cohorts, lack of standardized assays, and incomplete validation of prognostic value. Addressing these barriers is essential to enable effective patient stratification, early detection of therapeutic effects, and personalized interventions.

### Rationale

#### Importance of biomarkers in ALS

**FIGURE 1:**
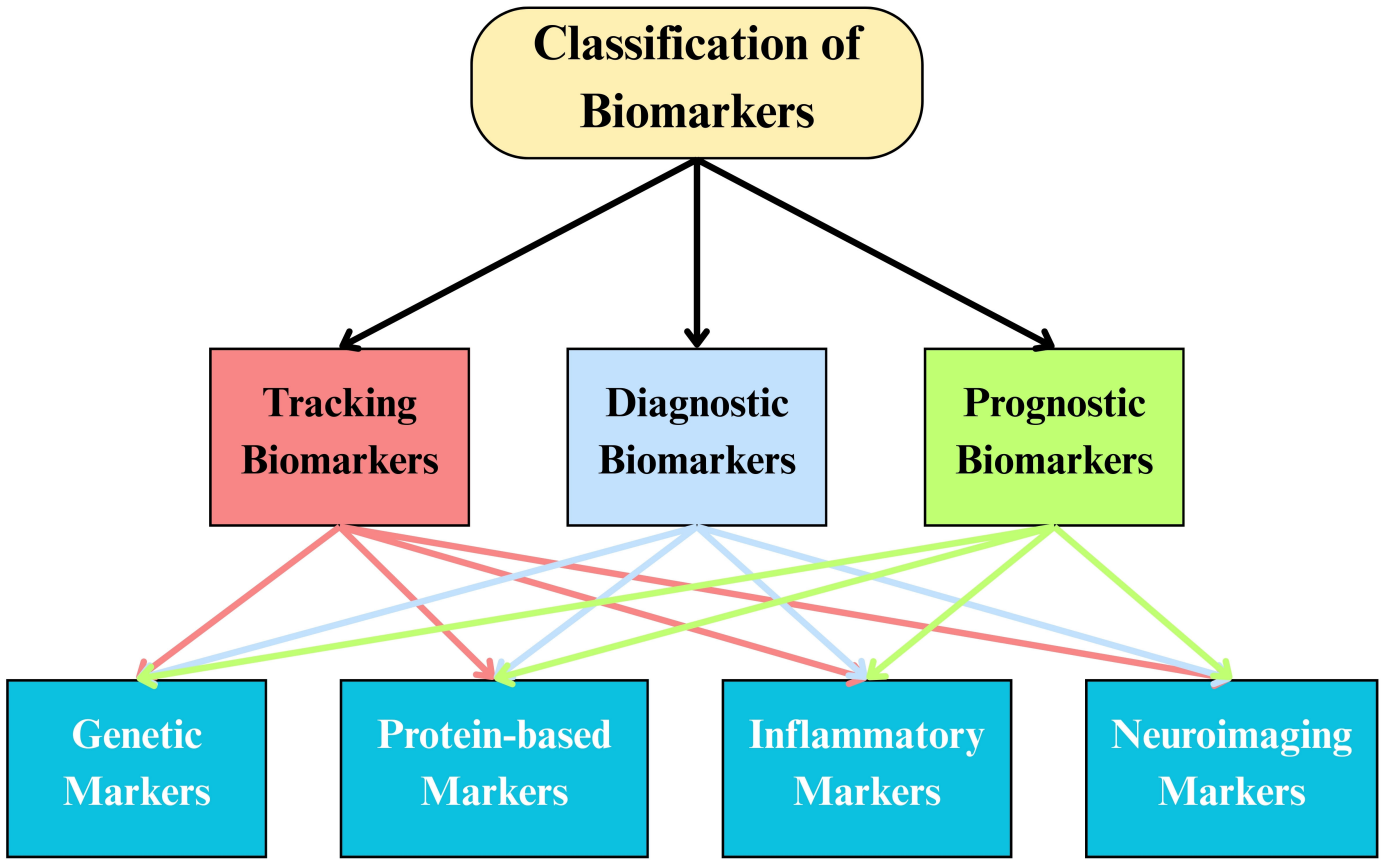
Classification and interoperability of biomarkers.

Biomarkers play an essential role in neurodegenerative disease research, offering measurable indicators of disease presence, activity, and treatment response. In ALS, biomarkers are typically classified by their diagnostic, prognostic, or pharmacodynamic value and include protein-based, genetic, inflammatory, and imaging markers (Figure 1) (Turner and Benatar, 2015). These tools not only facilitate understanding of the disease’s pathophysiology but also serve as surrogate endpoints in clinical trials, allowing earlier and more objective evaluation of therapeutic effects.

#### Neurofilament light chain (NFL) as a core biomarker

Among the biomarkers investigated in ALS, neurofilament light chain (NFL) has emerged as one of the most robust and clinically useful. NFL is a neuron-specific cytoskeletal protein released into cerebrospinal fluid (CSF) and blood following axonal damage (Bacioglu et al., 2016; Benatar et al., 2018, 2023). Elevated NFL concentrations are consistently observed in CSF, serum and plasma of ALS patients, correlating strongly with disease severity, progression rate, and survival (Benatar et al., 2018, 2023; Cebulla et al., 2023; Skillbäck et al., 2014; Steinacker et al., 2021). These characteristics make NFL a powerful prognostic and pharmacodynamic marker, now frequently employed as a secondary or surrogate endpoint in clinical trials to assess neuroprotective effects more rapidly than clinical outcomes alone (Gendron et al., 2017). The development of ultrasensitive assays has enabled reliable detection of NFL in peripheral blood, reducing the need for invasive lumbar punctures (Bacioglu et al., 2016) and facilitating its widespread adoption in studies such as the ongoing Lighthouse-II trial, where biomarker-based endpoints improve trial sensitivity and efficiency (Benatar et al., 2022).

#### Exploratory and emerging biomarkers

Beyond established markers like NFL, several exploratory biomarkers have gained attention for their potential to capture additional facets of MND pathophysiology. These include indicators of retroviral activation, immune and inflammatory dysregulation, metabolic dysfunction, and extracellular vesicle (EV)-associated cargo.

Retroviral biomarkers, particularly Human Endogenous Retrovirus-K (HERV-K), have been linked to aberrant reactivation in the central nervous system of ALS patients. Elevated HERV-K RNA and protein expression have been detected in cortical and spinal motor neurons, where they appear to drive neuroinflammatory responses and motor neuron toxicity (Dolei et al., 2019; Douville et al., 2011; Manghera and Douville, 2013). This has led to the exploration of antiviral and immune-modulatory therapeutic strategies (Li et al., 2022).

Extracellular vesicle associated biomarkers represent another promising class. Exosomes facilitate intercellular communication and can carry pathogenic proteins, microRNAs, and nucleic acids that reflect disease state (Doyle and Wang, 2019). In ALS, changes in exosome concentration, size distribution, and cargo composition, including increased TDP-43, *SOD1*, and miR-146a, have been reported (Anakor et al., 2021; Iguchi et al., 2016). Exosome-associated HERV-K RNA has also been proposed as a circulating biomarker linking retroviral activation and neuroinflammation.

Immune and inflammatory biomarkers such as interleukin-18 (IL-18), tumor necrosis factor-α (TNF-α), and CCL2 (MCP-1) have been associated with disease activity and rate of progression, emphasizing the contribution of neuroinflammation to ALS pathogenesis (Arru et al., 2021; Gille et al., 2019; Masrori et al., 2022). Recent findings highlight the prognostic utility of IL-18 when evaluated alongside NFL, reinforcing the value of multimodal inflammatory signatures (ALS Therapy Development Institute; Jiang et al., 2022).

In addition, metabolic and oxidative stress markers, including uric acid, creatinine, and oxidized lipid derivatives, have been explored as indicators of systemic alterations in cellular metabolism and mitochondrial function (Anand et al., 2013; Christidi et al., 2023; Fels et al., 2022; Kirk et al., 2019; Wang et al., 2020). These may provide complementary insights into peripheral mechanisms accompanying neurodegeneration.

Collectively, these exploratory biomarkers expand the current biomarker landscape in ALS, extending beyond neuronal injury to encompass immune, metabolic, and viral mechanisms. Their integration with established markers like NFL may ultimately yield a more comprehensive understanding of disease progression and therapeutic response.

### Research scope and objectives

This review synthesizes current evidence on biomarkers in ALS, with a particular focus on NFL and retroviral markers such as HERV-K. It examines their utility in improving patient stratification, enhancing clinical trial design, and informing therapeutic development. By critically assessing their role and limitations, the review aims to highlight how biomarker-driven approaches are reshaping ALS research toward more personalized and effective interventions. Biomarkers in ALS offer insights into diverse pathological mechanisms. Axonal injury can be assessed by neurofilaments (NFL, pNFH), while TDP-43 and phosphorylated tau reflect proteinopathy. Inflammatory cytokines (IL-6, TNF-α, IL-1, CRP) measure neuroinflammation, and genetic markers (*SOD1*, *C9orf72*) facilitate patient stratification. Together, these biomarkers can serve as secondary endpoints in clinical trials, providing quantitative measures of disease progression and mechanistic readouts of therapeutic interventions.

### Use of biomarkers in clinical trials

The integration of biomarkers has transformed the design and interpretation of ALS clinical trials by allowing earlier and more sensitive assessments of therapeutic response. Building on robust evidence linking elevated NFL to axonal damage and disease progression, biomarkers like NFL are now used as pharmacodynamic readouts in trials such as Lighthouse-II, where reductions in NFL levels may indicate treatment efficacy long before clinical outcomes are measurable (Fleming and Powers, 2012).

By integrating biomarker data into interim analyses, adaptive trials such as Lighthouse-II can refine cohort allocation, recruitment, and arm continuation based on emerging efficacy or futility signals. In ALS, such designs improve the quality and relevance of collected data by directing enrolment towards promising interventions and discontinuing unresponsive arms earlier. This approach enhances trial efficiency, accelerates assessment of therapeutic efficacy, and reduces patient exposure to ineffective treatments (Gold et al., 2019).

## Biomarkers in motor neuron disease and their role in clinical trials

Biomarkers are biological indicators used to diagnose and monitor diseases, assess disease progression, and evaluate treatment responses. In motor neuron disease, biomarkers play a critical role in advancing clinical and therapeutic understanding of the disease (Khabibrakhmanov et al., 2022). MND is characterized by the progressive loss of motor neurons, leading to severe physical impairment and often fatal respiratory complications. The urgency to develop effective treatments has spurred extensive research into biomarkers that can serve as objective, reliable measures of disease state and progression (Khabibrakhmanov et al., 2022; Masrori et al., 2022; Turner and Benatar, 2015). Currently, biomarkers in ALS encompass a variety of molecular, genetic, and imaging parameters, with a focus on those that correlate with disease severity and serve as surrogate or secondary endpoints in clinical trials (Arru et al., 2021; Turner and Benatar, 2015).

### Biomarkers as secondary endpoints in ALS

**FIGURE 2:**
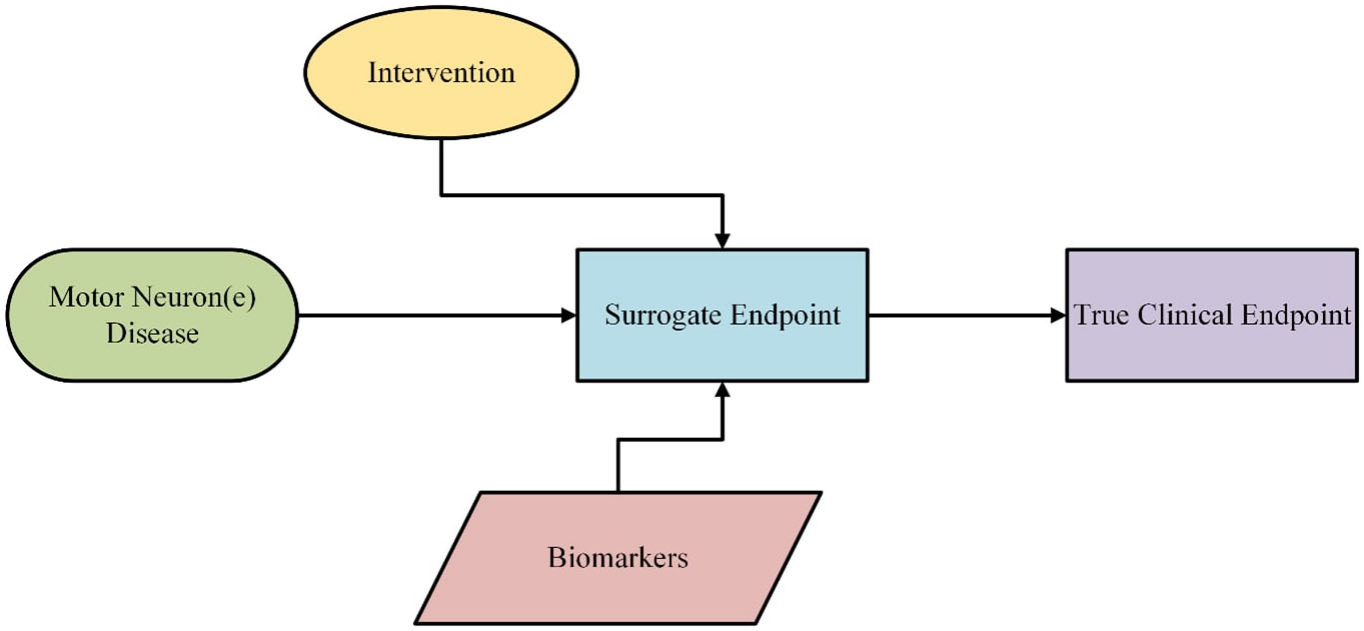
Biomarkers as surrogate endpoints in clinical trials.

Historically, ALS trials have relied on primary endpoints such as survival or decline in functional ability. However, these outcomes are often confounded by clinical heterogeneity and require long observation periods (Iguchi et al., 2016). Biomarkers offer complementary secondary endpoints that provide insight into subclinical disease activity, even before functional deterioration becomes apparent (Figure 2) (Steinacker et al., 2021).

Building on the strong association between elevated NFL and neurodegeneration, recent studies have successfully incorporated biomarker measures into trial outcomes. The MIROCALS trial, for instance, evaluated low-dose interleukin-2 (IL-2LD) as an adjunct to riluzole in ALS. While unadjusted survival outcomes were not significant, stratification by CSF phosphorylated neurofilament heavy chain (pNFH) revealed a survival benefit among patients with lower baseline pNFH levels (Bensimon et al., 2025). Additionally, IL-2LD treatment significantly increased regulatory T-cells (Tregs) and reduced plasma CCL2, demonstrating biological target engagement. Such studies underscore the potential of biomarker stratification to identify subgroups most likely to benefit from treatment and to uncover therapeutic signals that might otherwise remain undetected.

### Biomarkers in the design of clinical trials in ALS

Biomarkers now play a pivotal role in trial design, addressing long-standing challenges posed by the heterogeneity and rapid progression of ALS (Malaspina, 2024; Martinez et al., 2017; Taga and Maragakis, 2018). NFL, in particular, provides reproducible and quantifiable data that can stratify patients by disease stage or rate of progression (Benatar et al., 2023; Gendron et al., 2017). This facilitates more homogeneous trial cohorts and enables adaptive or enrichment strategies that improve statistical power and efficiency.

Adaptive trial designs informed by interim biomarker data allow for real-time protocol adjustments, such as altering sample size, refining endpoints, or modifying treatment arms, based on evolving biological evidence. This biomarker-driven, adaptive approach enhances trial efficiency by enabling real-time monitoring of treatment effects through markers such as NFL. In ALS, where progression is rapid and variable, this allows early identification of responders, informed dose adjustments, and adaptive modifications to trial design, such as reallocating participants or refining inclusion criteria. As demonstrated by the Lighthouse-II trial, this strategy accelerates the collection of meaningful data on therapeutic efficacy, reduces exposure to ineffective interventions, and shortens development timelines, making trials faster, more efficient, and more responsive to biological signals (Fleming and Powers, 2012; Turner et al., 2013).

### Limitations of current biomarkers in ALS clinical trials

Despite their promise, current biomarkers face several limitations. NFL, although highly sensitive to neuronal injury, lacks disease specificity: elevated levels are also seen in multiple sclerosis, Alzheimer’s disease, and traumatic brain injury (Steinacker et al., 2021). Biological and environmental factors can also influence NFL concentrations, introducing variability in longitudinal assessments (Benatar et al., 2018).

Similarly, while retroviral markers like HERV-K hold potential, their specificity and reproducibility remain uncertain (Dolei et al., 2019). Variability in HERV-K expression between patients, coupled with an incomplete understanding of its regulatory mechanisms, complicates its standardization as a clinical biomarker (Douville et al., 2011; Manghera and Douville, 2013).

Neurofilament light chain levels can fluctuate with environmental and biological factors. A 2024 study reported a 3.5-fold increase in plasma NFL and a 5.7-fold increase in CSF, NFL in an asymptomatic individual at risk for genetic prion disease after 6 weeks of oral minocycline treatment for a dermatologic condition (Gentile et al., 2025; Witzel et al., 2021). This elevation occurred without any signs of neuronal damage, suggesting that minocycline may impair the clearance of NFL from biofluids rather than causing neurodegeneration. In a 2023 randomized trial involving 63 ALS patients, treatment with rapamycin, an mTOR inhibitor, was associated with changes in immune cell populations (Mandrioli et al., 2023). While the study primarily focused on regulatory T cells, alterations in immune modulation could indirectly affect NFL levels.

Moreover, ALS’s inherent heterogeneity, spanning genetic, environmental, and phenotypic dimensions, means that no single biomarker can fully capture disease complexity. To address these challenges, multimodal approaches incorporating additional biomarkers as secondary endpoints are increasingly considered. These include immune and inflammatory markers (e.g., IL-18, TNF-α, CCL2), extracellular vesicle/exosome-associated proteins and RNAs (e.g., TDP-43, *SOD1*, miRNAs), and metabolic or oxidative stress indicators (e.g., uric acid, creatinine, oxidized lipids) (Manghera and Douville, 2013; Meeter et al., 2018). Combining these with established markers like NFL and retroviruses may provide a more comprehensive assessment of disease progression, improve patient stratification, and enhance the sensitivity of clinical trials to detect therapeutic effects (Benatar et al., 2022; Manghera and Douville, 2013; Meeter et al., 2018; ResearchGate, 2025).

## Methodology

### Search strategy

A systematic, unbiased literature search was conducted on 15th May 2025, using PubMed, Embase and putting the same search terms into Google Scholar. PubMed search was conducted using the terms {[(Motor Neuron(e) Disease*) OR (Amyotrophic Lateral Sclerosis*)] AND (Biomarkers)} AND [Clinical Trial(s)] with the headings exploded to include relevant subheadings including ALS. On Medline and Embase, search was conducted using the terms Amyotrophic Lateral Sclerosis, Motor Neuron Disease, Biomarkers and Clinical Trials. Google Scholar was searched with the terms “amyotrophic lateral sclerosis,” “motor neuron disease,” “biomarkers,” “clinical trials.”

No language or date restrictions were applied. Published conference proceedings were also included if they met inclusion criteria. The reference lists of each included result were also assessed for relevant results. Because the analysis was based on data from published articles (secondary data), ethical approval and written informed consent from individual participants for this study was not necessary.

A broad search was necessary given the heterogeneity within this research field. The search strategy was developed collaboratively between the authors. The search terms “optimization,” and “clinical endpoint” were not included in the final searches as their addition yielded fewer results. We applied no date restrictions to ensure that no relevant studies were overlooked. However, we anticipated that more recent publications would be more likely to meet our inclusion criteria.

### Study selection

**FIGURE 3:**
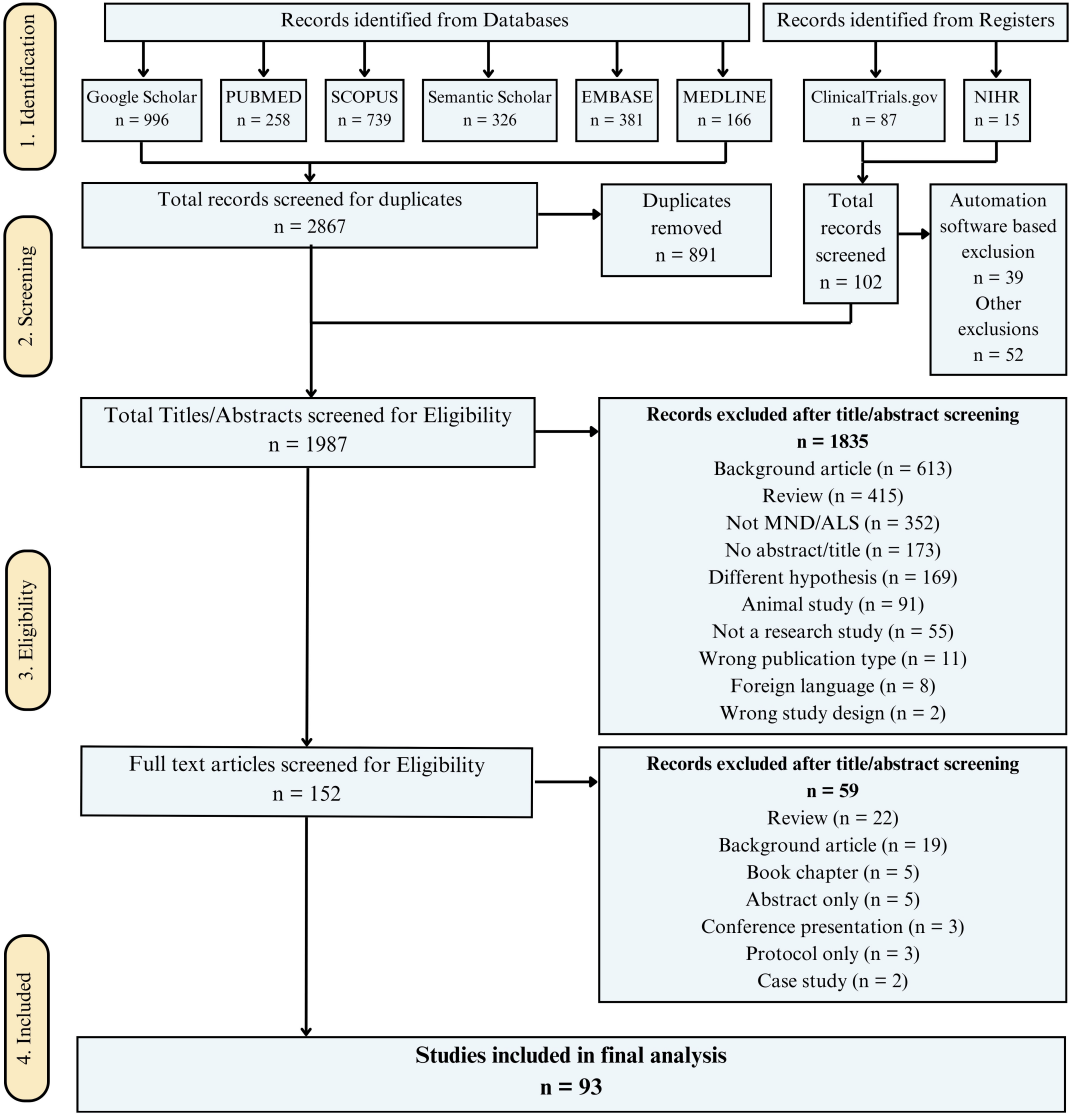
Screening and selection procedure using PRISMA guidelines (Anand et al., 2013). For more information, visit www.prismastatement.org.

A total of 152 studies were selected for full-text eligibility, out of w1hich 93 studies were included in the final analysis. The inclusion and exclusion criteria are detailed in Table 1 below. The PRISMA diagram for the screening process undertaken for the systematic review is shown in Figure 3.

**TABLE 1:** Inclusion and exclusion criteria.

Inclusion criteria	Exclusion criteria
1. Study type: a. Randomized controlled trials (RCTs), cohort studies, case-control studies, or observational studies involving clinical trials. b. Studies specifically assessing biomarkers related to ALS/MND in clinical trials, cohort or case-control studies, pilot/proof-of-concept investigations, and cross-sectional analyses. c. Studies that include longitudinal data assessing biomarkers for disease progression, prognosis, or therapeutic response.	1. Study scope: a. Studies focused solely on basic science, animal models, or *in vitro* biomarker studies without translation to human clinical trials. b. Studies that do not specifically address ALS/MND or focus on neurodegenerative diseases unrelated to ALS/MND.
2. Patient population: a. Studies involving human subjects diagnosed with ALS/MND (i.e., sporadic or familial forms). b. Inclusion of adult patients (≥ 18 years).	2. Biomarker focus: a. Papers on biomarkers not validated or utilized within a clinical trial or study design setting. b. Studies that do not provide data on the impact of biomarkers on clinical outcomes, disease progression, or treatment response.
3. Biomarker characteristics: a. Studies assessing biomarkers that are measurable in blood, cerebrospinal fluid (CSF), saliva, muscle biopsies, or other biological samples. b. Focus on neurofilament light chain (NFL), retroviral markers, inflammatory markers, genetic markers, or imaging biomarkers related to ALS/MND. c. Biomarkers utilized in clinical trials for patient stratification, disease monitoring, treatment efficacy, or surrogate endpoints.	3. Publication type: a. Case reports, conference abstracts, reviews, editorials, letters to the editor, or opinion pieces without original data. b. Non-peer-reviewed sources.
4. Publication type and language: a. Peer-reviewed journal articles. b. Full-text availability. c. Publications in English.	4. Duplicate or redundant data: Studies that are duplicates of previously published work or subsets of already included data (e.g., secondary analyses or interim results).
	5. Quality and methodological rigor: a. Studies with poor methodological quality (e.g., no clear outcome measures, lack of controls, small sample sizes without statistical significance). b. Exclude studies with insufficient details on biomarker measurement or lacking clinical relevance.

### Quality assessment

Quality assessment was performed using the QUADAS-2 (Quality Assessment of Diagnostic Accuracy Studies) tool. Each study was appraised to have high or low risk of bias in each domain. All studies which fitted inclusion criteria were included in the review regardless of risk of bias.

## Results

A total of 2,867 records were identified from databases (Google Scholar: 996; PubMed: 258; Scopus: 739; Semantic Scholar: 326; EMBASE: 381; MedLine: 166), along with 102 additional records from websites and registers (ClinicalTrials.gov: 87; Registers: 15). After removing 891 duplicates, 1,987 records remained for screening. Automation tools excluded 39 records, and 52 were excluded for other reasons. Following title and abstract screening, 1835 records were excluded, primarily due to being background articles (613), reviews (415), or unrelated to MND or ALS (352). A total of 152 full-text articles were assessed, with 59 excluded, leaving 93 studies for the final analysis, summarized in Figure 3.

This systematic review aimed to evaluate the efficacy and safety of various interventions for the treatment of amyotrophic lateral sclerosis (ALS) and motor neuron disease (MND) (Wang et al., 2020). A comprehensive search strategy was employed to identify relevant clinical trials, including those investigating pharmaceutical agents, novel therapeutic approaches, and complementary treatments. The included studies span a range of phases, from pilot trials to large, randomized, double-blind, placebo-controlled studies, each designed to assess different aspects of ALS treatment, including disease progression, survival rates, and biomarker alterations.

Among the diverse interventions explored, several trials have investigated novel drug candidates targeting specific molecular pathways implicated in ALS pathophysiology (Malaspina, 2024; Page et al., 2021). These included pharmaceutical-grade biotin (MD1003), which is proposed to enhance neuronal energy metabolism and support myelin synthesis (Juntas-Morales et al., 2020; Laursen et al., 2020); tauroursodeoxycholic acid (TUDCA), an anti-apoptotic and anti-endoplasmic reticulum stress agent that may protect motor neurons from programmed cell death (Albanese et al., 2022; Juntas-Morales et al., 2020; Lombardo et al., 2023), and Tecfidera (dimethyl fumarate), which activates the Nrf2 antioxidant pathway to reduce oxidative stress and neuroinflammation (Lombardo et al., 2023; Vucic et al., 2020).

In addition, several studies focused on immune-modulatory strategies, such as NP001 and the anti-CD14 antibody IC14, aimed at modulating systemic and neuroinflammatory processes implicated in ALS progression (Benatar et al., 2024; Forrest et al., 2024; Gelevski et al., 2023; Meininger et al., 2017; Vucic et al., 2020). Other trials evaluated neuroprotective agents, including ozanezumab and arimoclomol, which target pathways involved in neuronal survival and protein homeostasis (Benatar et al., 2024; Forrest et al., 2024; Gelevski et al., 2023; Meininger et al., 2017).

Finally, some studies assessed the role of adjunctive supportive therapies, such as non-invasive ventilation (NIV) and percutaneous endoscopic gastrostomy (PEG), which, although not disease-modifying, have been shown to improve survival and quality of life in ALS patients (Burkhardt et al., 2017). Importantly, many of these trials now incorporate biomarkers—such as neurofilament light chain, inflammatory cytokines, and exosomal markers—as secondary or exploratory endpoints, enabling more sensitive monitoring of treatment effects and providing mechanistic insights into how these interventions influence disease biology. This review also includes studies investigating promising biomarkers in ALS, such as NFL, and their association with disease progression and response to treatment (Skillbäck et al., 2014). A notable aspect of several trials was the exploration of different treatment regimens, including drug combinations, adaptive trial designs, and personalized medicine approaches, to better tailor interventions to individual patient needs. Trials like the MND-SMART study exemplify a pioneering multi-arm, adaptive approach in ALS/MND research. Unlike traditional single-intervention trials, MND-SMART evaluates multiple therapies within a single overarching framework, allowing interventions to be added, dropped, or modified based on interim efficacy and futility analyses. The design also uses shared placebo groups, reducing participant numbers and accelerating the assessment of promising treatments. By incorporating biomarker-based endpoints such as neurofilament light chain and inflammatory markers, the trial can detect biological effects earlier than clinical outcomes alone. This flexible, efficient framework represents a paradigm shift in ALS trial design, optimizing resources and patient benefit (Wong et al., 2022).

Given the complexity of ALS and the variability of patient responses, the results of these trials provide critical insights into the ongoing efforts to identify effective treatments for ALS (Table 2). In the following section, we present a detailed table summarizing the characteristics of the trials reviewed, including study design, scientific basis/rationale, outcomes assessed, and key findings.

**TABLE 2:** Biomarkers in amyotrophic lateral sclerosis (ALS): applications in clinical trials and disease monitoring.

Biomarker	Type	Clinical trial	Scientific basis/rationale	Details	References
Neurofilament light chain (NFL)	Protein	AP-101 Phase 2	Marker of axonal injury, reflects ALS progression	Monoclonal antibody targeting semaphorin 4D; plasma NFL measured as secondary endpoint	Neals
		PrimeC phase IIb	Marker of neurodegeneration	Ciprofloxacin + celecoxib; reduced NFL and slowed disease progression	Salomon-Zimri et al., 2023
		CNM-Au8 healy ALS platform	Reflects axonal damage and oxidative stress	CNM-Au8 enhances neuronal bioenergetics; reduction in NFL observed	Vucic et al., 2023; Writing Committee for the Healey Als Platform Trial, Shefner et al., 2025
		RESCUE-ALS, CENTAUR, MND-SMART	Surrogate marker of neuronal injury	NFL measured longitudinally to monitor progression and treatment response	Bowser et al., 2024; Paganoni et al., 2021; Vucic et al., 2021, 2023; Wong et al., 2022
Phosphorylated neurofilament heavy chain (pNFH)	Protein	MND-SMART, CENTAUR	Reflects axonal damage, associated with disease progression	Used as secondary endpoint to monitor treatment response	Bowser et al., 2024; Paganoni et al., 2021; Wong et al., 2022
TDP-43 (CSF)	Protein	Verdiperstat trial	Targets TDP-43-associated neurotoxicity	Myeloperoxidase inhibitor; CSF TDP-43 measured to monitor treatment	Biohaven Pharmaceutical Holding Company Ltd, 2022; Writing Committee for the Healey Als Platform Trial, Shefner et al., 2025
BDNF	Protein	NurOwn phase II	Supports motor neuron survival and repair	Mesenchymal stem cells engineered to secrete BDNF; CSF BDNF measured	Berry et al., 2019
MicroRNA-206	Non-coding RNA	miRNA-206 Biomarker Study	Regulates gene expression, may influence ALS pathogenesis	Evaluated as prognostic biomarker and therapeutic target	Ricci et al., 2018; Waller et al., 2017
Tregs, CSF-pNFH, CCL2	Protein	MIROCALS Phase 2b	Immune modulation; axonal injury marker	IL-2LD increased Tregs, reduced CCL2; stratification by CSF-pNFH revealed survival benefit	ALS Therapy Development Institute; Bensimon et al., 2025; ResearchGate, 2025
Progranulin	Protein	AL001 Phase 2	Deficiency causes lysosomal dysfunction	AL001 increases progranulin levels; impact on ALS progression assessed	Health Research Authority
Inflammatory markers (IL-1, IL-6, TNF-α, CRP)	Protein	CENTAUR, MND-SMART, TUDCA-ALS	Reflect neuroinflammation linked to ALS progression	Plasma/CSF cytokine levels monitored to assess treatment effects	Albanese et al., 2022; Bowser et al., 2024; Fels et al., 2022; Lombardo et al., 2023; Paganoni et al., 2021; Wong et al., 2022
Urate	Metabolite	Radicava/edaravone	Neuroprotective properties; correlates with function	Blood urate levels monitored to evaluate relationship with disease progression	Berry et al., 2021
Lactate, pyruvate	Metabolite	Metabolomics-based study	Energy metabolism dysregulation in ALS	Longitudinal evaluation of metabolic biomarkers to track progression and therapeutic response	Abhinav et al., 2014; Kirk et al., 2019
Glycans	Protein modifiers	Glycan biomarker study	Glycosylation affects protein function and cellular stress	Specific glycan profiles measured as potential diagnostic/prognostic markers	Edri-Brami et al., 2012
Phosphorylated tau (pTau)	Protein	pTau biomarker analysis	Tau phosphorylation implicated in neurodegeneration	CSF and plasma levels assessed to differentiate ALS from other neurodegenerative disorders	Grossman et al., 2014
*SOD1*	Protein	Tofersen (ATLAS), VALOR	*SOD1* mutations cause familial ALS; therapeutic target	Monitored to assess efficacy of antisense oligonucleotides targeting mutant *SOD1*	Benatar et al., 2022; Miller et al., 2022
*C9orf72* repeat expansions	Genetic	FOCUS-C9, Tofersen (ATLAS), MND-SMART	Genetic cause of ALS/MND; target for RNA-based therapies	Used for patient stratification and monitoring therapy targeting repeat expansions	Benatar et al., 2022; Health Research Authority; Wong et al., 2022
Electrophysiological markers (e.g., CMAP, MUNE)	Electrophysiology	MND-SMART, CENTAUR	Reflect motor neuron function and degeneration	Measured longitudinally to track progression and therapeutic effects	Bowser et al., 2024; Paganoni et al., 2021; Wong et al., 2022
MRI imaging	Imaging	MND-SMART, CENTAUR	Brain atrophy and structural changes reflect neurodegeneration	MRI used to monitor disease progression and evaluate regional brain volume changes	Bowser et al., 2024; Paganoni et al., 2021; Simon, 2018; Wong et al., 2022
PET imaging	Imaging	MND-SMART, CENTAUR	Metabolic activity changes in CNS	PET used to assess neuroinflammation and metabolism	Bede and Pradat, 2019; Bowser et al., 2024; Paganoni et al., 2021; Wong et al., 2022
Creatine monohydrate	Metabolite	Phase 1b/IIa clinical trial	Supports mitochondrial function, may protect motor neurons	Monitored for effects on muscle strength and motor function	Imamura et al., 2019, 2022, 2019
High-dose biotin (MD1003)	Protein	Pilot study	Supports myelin and axonal integrity	Evaluated for potential slowing of ALS progression	Juntas-Morales et al., 2020
Ozanezumab (anti-serum amyloid)	Protein	Phase 2 trial	Targets amyloid proteins, may protect motor neurons	Assessed for impact on survival and functional outcomes	Meininger et al., 2017

## Discussion

This systematic review provides a comprehensive synthesis of 93 studies evaluating interventions in ALS and MND, with a particular focus on biomarkers that inform disease progression, prognosis, and therapeutic efficacy. ALS is a heterogeneous neurodegenerative disorder characterized by progressive loss of upper and lower motor neurons, leading to paralysis and respiratory failure. The rapid progression and clinical variability of ALS pose substantial challenges for clinical trial design and therapeutic development, highlighting the critical need for biomarkers that can serve as reliable secondary endpoints, facilitating earlier and more sensitive detection of treatment effects.

Among the most robust and consistently studied biomarkers, NFL has emerged as a central tool in both observational studies and clinical trials. NFL is a neuron-specific cytoskeletal protein released into the CSF and blood following axonal injury. Across multiple trials, including RESCUE-ALS, CENTAUR, and MND-SMART (Bowser et al., 2024; Paganoni et al., 2021; Vucic et al., 2021, 2023; Wong et al., 2022), elevated NFL levels have been shown to correlate strongly with faster disease progression, higher functional decline, and reduced survival, confirming its potential as a prognostic biomarker. NFL is increasingly used as a secondary endpoint in clinical trials, enabling researchers to monitor neuronal injury quantitatively, independent of clinical symptom fluctuations. For instance, in trials investigating NP001 and sodium phenylbutyrate, NFL levels allowed early assessment of therapeutic effects, providing a mechanistic readout of drug efficacy (Bowser et al., 2024; Forrest et al., 2024; Witzel et al., 2022). Similarly, pNFH reflects axonal damage and has been applied in trials such as MND-SMART and CENTAUR (Bede and Pradat, 2019; Ganesalingam et al., 2013; Lombardi et al., 2019; Paganoni et al., 2021; Witzel et al., 2022; Wong et al., 2022), demonstrating a positive correlation with disease progression rate and reinforcing its role as a secondary endpoint.

TDP-43, a hallmark proteinopathy in ALS, provides mechanistic insights into neurodegeneration (Bowser et al., 2024; Miller et al., 2022; Paganoni et al., 2021; Ricci et al., 2018; Verber et al., 2019). Elevated CSF TDP-43 levels are associated with more aggressive disease phenotypes, and while its use as a secondary endpoint is still exploratory, it offers a valuable readout in trials targeting protein aggregation and neuroinflammation. *SOD1*, central to familial ALS, has been effectively leveraged in genetic-targeted therapies such as Tofersen (ATLAS), with longitudinal measurements of mutant *SOD1* allowing evaluation of therapy engagement and functional outcomes (Bensimon et al., 2025; Bowser et al., 2024; Miller et al., 2022).

Genetic biomarkers also play a pivotal role in patient stratification. *C9orf72* repeat expansions, the most common genetic cause of familial ALS, do not fluctuate dynamically with disease progression but are crucial for enrolling genetically defined cohorts and interpreting treatment responses. Trials such as FOCUS-C9 (Christidi et al., 2023; Ejebe et al.; Health Research Authority) and MND-SMART have used *C9orf72* genotyping to identify patients most likely to benefit from targeted therapies, facilitating the use of secondary endpoints like NFL, pNFH, and inflammatory biomarkers within genetically homogeneous subgroups.

Neuroinflammation is a key pathophysiological feature in ALS, and inflammatory biomarkers such as IL-6, TNF-α, IL-1, and CRP have been extensively studied (Belge et al., 2002; Berry et al., 2019; Bowser et al., 2024; Witzel et al., 2022). Observational studies consistently report that elevated cytokine levels correlate with faster functional decline. In clinical trials like MND-SMART and TUDCA-ALS (Fels et al., 2022; Fleming and Powers, 2012; Juntas-Morales et al., 2020; Lombardi et al., 2019; Lombardo et al., 2023; Wong et al., 2022), these markers were used as secondary endpoints to quantify immune modulation and therapeutic effects, offering mechanistic insights into the efficacy of anti-inflammatory or immunomodulatory treatments. The MIROCALS trial illustrates this principle: stratifying patients based on CSF-pNFH and regulatory T-cell (Treg) levels revealed a survival benefit in those with lower axonal damage and enhanced immune regulation, demonstrating how combining inflammatory and axonal biomarkers can improve patient selection and interpret treatment outcomes (Bensimon et al., 2025; Imamura et al., 2022).

Imaging biomarkers, including MRI and PET, provide non-invasive measures of neurodegeneration. Longitudinal MRI studies correlate regional brain atrophy with clinical progression (Bede and Hardiman, 2018; Bede et al., 2019; Belge et al., 2002; Berry et al., 2021; Fels et al., 2022), while PET imaging can detect metabolic and neuroinflammatory changes (Bede and Pradat, 2019; Paganoni et al., 2021; Salomon-Zimri et al., 2023; Wong et al., 2022). These imaging measures serve as secondary endpoints in trials, enabling early detection of structural or functional responses to therapy, complementing fluid-based biomarkers such as NFL or cytokines.

Electrophysiological biomarkers, including CMAP and motor unit number estimation (MUNE), are highly sensitive to motor neuron loss and functional decline. These measures have been applied as secondary endpoints in trials including CENTAUR and MND-SMART (Bede and Pradat, 2019; Bowser et al., 2024; Paganoni et al., 2021; Witzel et al., 2022; Wong et al., 2022), providing quantitative tracking of disease progression independent of clinical rating scales.

Metabolic biomarkers, including urates, lactate, and pyruvate, have also demonstrated utility. Elevated urate levels correlate with slower ALS progression in observational studies (Takahashi et al., 2022a,b; Verber et al., 2019), supporting their use as secondary endpoints in trials assessing neuroprotective interventions. Similarly, longitudinal measurements of lactate and pyruvate reflect energy metabolism dysregulation, a key pathological feature in ALS (Abhinav et al., 2014; Ejebe et al.; Kirk et al., 2019; Mehta et al., 2019), and may serve as mechanistic readouts of treatment efficacy.

Additional exploratory biomarkers, such as BDNF, progranulin, microRNAs, glycan modifications, and pTau, have been investigated in early-phase studies (Abhinav et al., 2014; ALS Therapy Development Institute; Berry et al., 2019; Ejebe et al.; Grossman et al., 2014; Ricci et al., 2018; Waller et al., 2017). While correlations with progression rates remain under evaluation, these biomarkers are increasingly integrated as secondary endpoints to assess biological responses to novel therapeutics, especially those targeting neurotrophic support, lysosomal function, or protein homeostasis.

The integration of these biomarkers in clinical trial design represents a paradigm shift in ALS research. Traditional endpoints, such as survival or functional rating scales, are often limited by disease heterogeneity and the slow emergence of observable changes. By incorporating biomarkers with demonstrated correlations to progression, trials can detect treatment effects earlier, stratify patients according to risk or disease stage, and provide mechanistic insight into drug action. For example, the combined use of NFL, pNFH, and inflammatory markers has enabled adaptive trial designs, such as MND-SMART, where interim biomarker analysis informs modifications in trial arms and patient allocation (Bede and Pradat, 2019; Bowser et al., 2024; Paganoni et al., 2021; Witzel et al., 2022; Wong et al., 2022).

Despite these advances, challenges remain. Many biomarkers, including inflammatory cytokines and exploratory molecules, lack standardized measurement protocols, limiting comparability across studies. Biomarker levels can also vary due to comorbidities, environmental factors, or genetic background, complicating interpretation. Additionally, while NFL and pNFH show strong associations with progression, other biomarkers, such as BDNF or glycan modifications, require further longitudinal validation to confirm their prognostic value. Addressing these limitations will require harmonized assays, multicentre validation, and integration of multimodal biomarker panels to fully capture disease heterogeneity and therapeutic response.

## Future work

Future work in ALS research must prioritize the discovery, validation, and optimization of new biomarkers that can guide both early diagnosis and therapeutic monitoring. While several biomarkers, such as NFL have shown promise in tracking disease progression, further efforts are needed to establish more reliable and sensitive markers for different stages of ALS and for predicting patient response to therapy. The identification of novel biomarkers that can capture distinct pathophysiological processes, such as neuroinflammation, mitochondrial dysfunction, and axonal damage, is essential for advancing personalized medicine in ALS.

One key area of future research is the exploration of genetic biomarkers, particularly for rare genetic subtypes like *C9orf72* and *SOD1* mutations. These genetic biomarkers are essential for stratifying patients in clinical trials, ensuring that specific therapies targeting these mutations are tested in the appropriate patient populations. Investigating genetic signatures of ALS will also help uncover novel therapeutic targets and guide the development of gene therapies. Additionally, epigenetic markers could provide deeper insights into how environmental factors and lifestyle influence disease progression and response to treatment (Figueroa-Romero et al., 2012; Griñán-Ferré et al., 2024; Jimenez-Pacheco et al., 2017).

Immunological biomarkers also warrant increased attention, given the growing evidence of neuroinflammation playing a critical role in ALS pathogenesis. Biomarkers such as pro-inflammatory cytokines (e.g., IL-6, TNF-α) (Bowser et al., 2024; Gille et al., 2019; Jiang et al., 2022) and neuroinflammatory proteins could aid in identifying patients who would benefit from immunomodulatory treatments. Future trials should focus on testing the predictive value of these biomarkers in personalized therapies aimed at reducing neuroinflammation.

Multi-biomarker panels may be the key to improving clinical trial outcomes. Rather than relying on a single biomarker, combining multiple biomarkers—such as NFL, pNFH, and inflammatory markers (Ganesalingam et al., 2013; Jiang et al., 2022; Manghera and Douville, 2013; Upadhyay et al., 2016) could provide a more comprehensive picture of disease activity and treatment efficacy. Future trials should incorporate such panels to capture different dimensions of ALS pathology and enhance the precision of outcome measures.

To optimize clinical trials using these biomarkers, adaptive trial designs should be employed. Multi-arm adaptive trials, such as the MND-SMART (Wong et al., 2022) study, allow for the simultaneous testing of multiple therapies while incorporating real-time biomarker data to adjust the treatment regimens based on patient response. This approach not only speeds up the trial process but also maximizes the likelihood of identifying effective treatments. Moreover, the integration of biomarker-based endpoints (Chipika et al., 2019; Fleming and Powers, 2012; Menke et al., 2017; Staffaroni et al., 2019) in trial designs, alongside traditional clinical endpoints, will ensure that trial results more accurately reflect the disease mechanisms and therapeutic benefits.

## Data Availability

The original contributions presented in this study are included in this article/supplementary material, further inquiries can be directed to the corresponding authors.
